# Population characteristics and predation rates of the dominant soft-bodied and durophagous predators on temperate intertidal shores

**DOI:** 10.1098/rsos.240308

**Published:** 2024-06-19

**Authors:** Lloyd S. Peck, Hannah E. Mance, Miles B. Ellis, Daniel Matok, Laura J. Grange

**Affiliations:** ^1^ British Antarctic Survey, Natural Environment Research Council, High Cross, Madingley Road, Cambridge CB3 0ET, UK; ^2^ School of Ocean Sciences, Bangor University, Bangor LL57 2DG, UK

**Keywords:** predation, predator/prey, food web, energy flow, sessile, benthic

## Abstract

Substantial research exists on predation and its ecology. Most research has focused on durophagous fishes, brachyuran crabs, and lobsters. Data are lacking, however, on soft-bodied predators like anemones, and their contribution to overall levels of predation remains largely unevaluated. Here, we compared predation rates of the durophagous predator, the crab *C. maenas* and the soft-bodied predator, the anemone *Actinia equina* on 15 intertidal shores around Anglesey, north Wales, UK. We employed a novel approach to assess predation based on measuring faecal output from recently collected individuals and converting it to food consumed using absorption efficiencies (AEs) measured using potential prey species inhabiting the same shores. Anemone mean abundance was 8.21 (± 0.27, s.e.) individuals.m^−2^, whereas for *C. maenas* it was 0.23 (± 0.02, s.e.) individuals.m^−2^. AEs when fed mussel tissue, a polychaete worm, or a shrimp were 92.8–94.0% in *C. maenas* and 40.5–95.8% in *A. equina*. This difference in values reflected the different feeding modes of the two predators. Unexpectedly, *A. equina* consumed 3.5–7 times more prey than *C. maenas*. The consumption of larger amounts of prey by an anemone than the dominant durophagous predator has important consequences for calculating energy flows in food webs, understanding predation controls in assemblages, and potentially for wider predation trends.

## 1. Introduction

It has been recognized for nearly a century that predation is a fundamental process impacting or structuring food webs and ecosystems [1]. Debate has been intense for over 50 years as to its importance as a top–down controlling factor and the prevalence of trophic cascades as opposed to bottom–up control of food webs [2–4]. The intertidal has been the focus of much ecological research because of its ease of access and varied environment. Predation in the intertidal has been the subject of intense study for over 50 years [5,6]. Some major advances in understanding have come out of work on intertidal assemblages, including the effects of the removal of predators followed by intense downstream grazing by herbivores that changes the structure of ecosystems [7]. Further to this, there is evidence that environmental alteration owing to climate change is impacting the roles of top–down and bottom–up processes in marine ecosystems [8].

A very large proportion of the above-mentioned research has focussed on data collected on predators with hard skeletons that either crush their prey or access tissues via drilling through exoskeletons, and their attacks are often obvious owing to physical signs on prey skeletons (e.g. [9,10]). Most analyses of large-scale trends in predation across geographical regions have also primarily been based on durophagous predators (e.g. [11,12]). There is, however, another class of predators that has received much less attention, where there are no observable traces on prey skeletons. This is predation by soft-bodied predators such as anemones, starfish, various soft and hard corals and some worms such as the giant engulfing nemertean *Parborlasia corrugatus* [13]. The contribution of such predators to overall levels of predation in assemblages is hard to quantify, but several instances have been noted where they have had very powerful impacts, such as during outbreaks of the crown of thorns starfish on coral reefs [14,15], or where the effect of predator removal was assessed [16]. In the intertidal, the importance of soft-bodied predators has also been noted from the effects of mass mortalities in starfish, which have been used to demonstrate predators do exert top–down control over prey population sizes, and set lower distribution limits [17]. Even here, however, work has been primarily focused on asteroids. Furthermore, few, if any, studies have assessed predator impacts in terms of numbers of prey or biomass taken by predators, an essential requirement for building food webs and ecosystem models.

Substratum type affects the composition of predators inhabiting a shore. Most engulfing soft-bodied predators live on hard substrata either attached, as in anemones, or moblile, as in starfish. Some soft-bodied species do inhabit sediments, including burrowing anemones that occur in all oceans from the tropics to the poles [18,19], but they are predominantly a taxon attached to hard substrata [20], although their distributions are affected by a range of factors [21]. Crabs, on the other hand, are common on both hard and soft substratum shores, though they are characterized by seeking refuge from predators, especially birds during emersion [22], and species inhabiting soft sediments often use burrows as refuges. Other factors than substratum affect the distributions and densities of crabs and anemones, and these include shore exposure [21,23,24], sand abrasion [25], and competition [26]. There are thus many factors that will affect the eventual predation rate of any given intertidal predator, and information is lacking on the relationship between how factors combine to control predator densities and their performances.

In this study, we aimed to address this lack, or absence, of data on levels of predation by soft-bodied predators by estimating feeding rates in the most common soft-bodied predator, the anemone *Actinia equina,* on 15 beaches around the Isle of Anglesey, north Wales, UK, and comparing it to the most common durophagous predator, the crab *C. maenas*. There was a further aim to assess populations present in relation to the substratum and exposure characteristics of sites. We used a novel approach to quantify the amounts of prey taken. This was achieved by measuring faecal egestion (FE) in recently collected individuals combined with absorption efficiency (AE) assessments measured by feeding the two predators on representative prey items present on the beaches studied. This allowed amounts eaten to be estimated from faecal production by an individual or given biomass of predator. Combining this with predator population density and biomass estimates from the shore allowed the estimation of the amount of prey consumed by populations of target predator species in the days before collection. Faecal production has been used in the past to assess, e.g. seasonal patterns in feeding activity in primary consumers (e.g. [27,28]), in grazers (e.g. [29]), and in scavengers and predators (e.g. [13]). FE rates have also been used to assess field feeding rates in Antarctic limpets [30]. We are not, however, aware of any studies quantifying levels of predation by marine predators using this approach.

## 2. Material and methods

### 2.1. Field surveys

**Figure 1.**
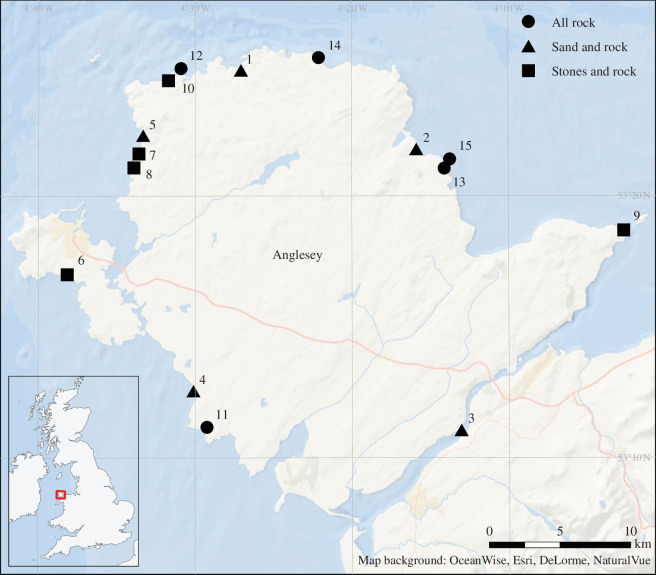
Survey sites around Anglesey, north Wales, UK, where predator abundances were assessed. Symbols refer to substratum type (circles = all rock; squares = stones and rock; triangles = sand and rock). Site details, name, latitude, longitude, substratum type and exposure rating are given in the electronic supplementary material, table S1.

Census surveys were made of the dominant intertidal rocky shore crustacean (the brachyuran crab *C. maenas*) and soft-bodied (the anemone *A. equina*) predators around the coastline of the Isle of Anglesey, north Wales, UK. Surveys were made between the 22 June and 10 July 2021. During this period, daily maximum tidal heights ranged from 4.56 m (4 July) to 5.75 m (25 June). Maximum air temperatures ranged from 16°C to 21°C with the exception of 3 days where they were 14°C (22 June), 13°C (26 June) and 12°C (24 June). Minimum air temperatures ranged from 10°C to 14°C with the exception of 2 days when they were 7°C (24 June) and 4°C (23 June). There was precipitation on 22–26 June, 28 June and 3–7 July. Winds were light across the whole period. Fifteen intertidal sites were identified as suitable on the basis of accessibility from land, substratum type, width (>20 m wide), a distance of 20–60 m between low and high water marks and the presence of at least five rock pools (figure 1). Three substratum types (all rock, rock with stones, and rock with sand; *n* = 5 each) were chosen for study, and exposure ratings for each site following [24,31] were also calculated (electronic supplementary material, table S1).

At each site, at low tide, three transects were set out 10 m apart from the high tide level to the low water mark. Each transect had 20 equidistant points marked (distance between points varied with transect length), and at each point, quadrats of 0.25 m^2^ area were laid out 1 m distance on either side of the transect line. A total of 40 quadrats were therefore assessed on each transect, and 120 were measured per site. Quadrats were classified as either dry or wet if more or less than 5 cm depth of water was present over more than half the quadrat area. Evaluations were made beginning at the low water mark and working towards the high water mark, and all individuals present in each quadrat of each species were recorded.

Visual surveys were first conducted. Where there were rocks with overhangs or crevices, manual checks were carried out, and where small- or medium-sized rocks were present, these were turned over. The size of each individual (basal diameter of *A. equina* and carapace width for *C. maenas*) was measured using Vernier callipers (± 0.1 mm). Animal weight (wet weight, dry weight and ash-free dry mass (AFDM)), and hence population biomass, were estimated from weight-to-linear dimension relationships obtained from collected samples across the size range present (see §3.1). Sex of crabs and colour morph of anemones were not recorded.

### 2.2. Faecal egestion

For both species, 30 individuals across a wide size range (5–40 mm diameter for *A. equina* and 5–60 mm carapace width for *C. maenas*) were used as a measure of size in FE experiments. All the anemones were collected from Porth Nobla (figure 1; site 4, 53°12′38″ N, 04°30′11″ W), and 25 *C. maenas* were collected from the same site, with five more collected from St George’s Pier, Menai Bridge (53°13′32″ N, 04°09′36″ W). All specimens were collected at low tide and from close to the mid-tide level. After collection, they were transferred to the Craig Mair aquarium, Bangor University in insulated boxes within an hour of collection. Anemones were removed from rock surfaces using a thin, blunt plastic spatula and crabs were hand-collected.

In the aquarium system, anemones and crabs were held in plastic tanks of 1– 5 l volume, depending on the size of the individual. Each tank was fitted with a fine mesh net cover to ensure specimens stayed *in situ*. Tanks were also weighted with stones and submerged in 500 l volume flow-through water baths with water flows across experimental tanks of 10 cm to 20 cm s^−1^ to maintain temperatures close to local seawater values and to allow high levels of water and gas exchange between the water baths and tanks. Additionally, 80% of the water in each tank was exchanged daily to ensure high water quality conditions. Conditions in the system were close to external ambient (18–20°C and 31–34 Practical Salinity Units (PSU)) throughout the experiments, and the light regime employed was ambient daylength. On being placed into tanks, anemones rapidly attached to either the tank wall or the stone weight, and all individuals were attached within 2 h. Crabs generally maintained positions around the base of stone weights but also moved around the tanks. Observations of anemones and crabs showed similar responses to external stimuli and movements around tanks as individuals held nearby in large tanks with constant high water flow and aeration.

All faeces produced were collected by pipette in 24 h intervals after specimens had been placed in tanks. Following collection, faeces were briefly rinsed with fresh water to remove salts and transferred to pre-ashed, pre-weighed aluminium boats. Excess water was removed by pipette, and the faeces were weighed to give a wet weight. They were then dried at 60°C for 24 h and reweighed before being ignited in a muffle furnace at 475°C for 12 h. Samples were allowed to cool in the furnace until they were at 50°C when they were once again weighed. Differences between ash weight following ignition at 475°C and dry weight provided AFDM, an estimate of organic content. All weight measurements were carried out using a Sartorius A120S analytic balance (±0.0001 g).

After animals had ceased producing faeces, they were killed by being wrapped in insulating material and placed in a freezer at −20°C. Specimens were checked at 10 min intervals, until there was no response to external stimuli. They were then processed as for faeces to obtain wet weight, dry weight and AFDM estimates. FE was calculated as g AFDM faeces.d^−1^. g animal AFDM^−1^.

### 2.3. Predator biomass

For both species, 30 specimens were collected across a wide size range (see §2.1). Individuals were first measured for linear dimension as in §2.1. They were then surface-dried using paper towels and weighed using a Sartorius A120S analytic balance (± 0.0001 g) to provide an estimate of live weight. Following measurement of live weights, specimens were transferred to pre-ashed, pre-weighed aluminium boats and dried and ignited in a muffle furnace as in §2.2 to obtain estimates of individual AFDM. The biomass of the mean-sized individual in field populations was calculated for each species from relationships of ln AFDM versus ln diameter, and the total field biomass was then obtained from the number of individuals present.

AFDM was chosen as the metric for comparison between the species for two reasons. Firstly, it represents the amount of metabolizing tissue in the animal. Secondly, other measures include obvious substantial factors making comparisons difficult, as live weight includes much larger amounts of water in the anemones and hard skeleton in the crabs and dry weight includes a very large proportion of hard skeleton in the crabs not in the anemones. AFDM should represent the same thing in both species, the equivalent of dry tissue weight often used in measuring the size of metabolically active tissue or organic tissues in many studies.

### 2.4. Absorption efficiency

Specimens of each species (*n* = 30) were collected and returned to the laboratory where they were held individually in containers of 1–5 l capacity, depending on the size of the specimen. These containers were covered with a fine mesh (to ensure animals did not escape) and were submerged in larger 500 l tanks that were supplied with filtered, flowing ambient seawater. Animals were starved for 7 days to clear their guts. They were then fed weighed amounts of prey that occur in the local environment and are representative of the potential types of food available (the shrimp *Crangon crangon*, soft tissues of the mussel *Mytilus edulis* and the polychaete ragworm *Hediste diversicolor*) at approximately 5–10% wet body weight. After 24 h, any remaining food was removed and weighed. Representative samples of each food species of approximately the same size as those offered to experimental specimens and all remaining food items after 24 h were assessed for dry weight and AFDM as above. After feeding, faeces were collected as previously at 24 h intervals, and faecal wet weight, dry weight and AFDM were measured. AE was calculated as:


$$AE\left(\%\right)=\left(\frac{C-F}{C}\right)\times 100,$$

where C is the weight of food consumed and F is the weight of faeces produced. It can be expressed as wet weight, dry weight or AFDM.

Measuring AE in 30 animals fed a diet of species representative of the organic composition of the types of animals probably consumed in the wild allows for good estimates of the conversion of prey to faeces. Obtaining estimates of daily faecal production by 30 wild-caught individuals over the days immediately after collection allows daily faecal production by an average individual in the population to be estimated (see §2.2). Combining daily faecal production by an average individual with estimated AE allows estimates of amounts of food consumed by an average individual to be made. Multiplying this by population density measurements allows for food consumed by the whole population to be estimated.

### 2.5. Data analysis

#### 2.5.1. Predator densities

All data were analysed in RStudio (RStudio Team, 2020; v. 1.4.1106). Neither abundance (Shapiro–Wilk, anemones: *W* = 0.689, crabs: *W* = 0.230, *p* < 0.05) nor predator size data (Shapiro–Wilk, anemones: *W* = 0.976, crabs: *W* = 0.854, *p* < 0.05) were normally distributed. Non-parametric analyses were, therefore, conducted. Kruskal–Wallis one-way analyses of variances were conducted for both species, to assess differences in abundance and size between all survey shores, substratum type and exposure. Post hoc Wilcoxon rank-sum tests were conducted if significant differences were reported. Abundance and size data were then subset by survey shore substratum type and shore exposure, and Kruskal–Wallis tests were conducted within each subset to investigate differences in predator abundances and size between wet and dry sampled areas.

#### 2.5.2. Relative predation rates

FE rates and AEs were used to compare *in situ* predation rates for both species, by multiplying the calculated abundances of animals at different shores with the FE values. Predation rate was calculated as follows:


$$Predation\;\;rate\;\;m^{-2}=(FE/AE).mean\;\;animal\;\;weight\;\;(g).predator\;\;abundance\;\;\left(m^{-2}\right)$$

Predation data were expressed as g AFDM.m^−2^.d^−1^. All data errors quoted are ± standard error (s.e.).

## 3. Results

### 3.1. Predator densities, size frequencies and biomass

Across the 15 sites studied, a total of 450 m^2^ of substratum was surveyed and 14 772 *A*. *equina* and 416 *C. maenas* were recorded. Average densities were 8.21 ± 0.27 (s.e.) individuals.m^−2^ (range = 0−100 individuals.m^−2^) for *A. equina* and 0.23 ± 0.02 individuals.m^−2^ (range = 0–12 individuals.m^−2^) for *C. maenas*. Details of abundances at each site surveyed are given in the electronic supplementary material, table S2. The highest average abundance for *A. equina* in any bay occurred in Cemlyn Bay (16.73 ± 1.74 individuals.m^−2^), a site with all rock substratum, and Porth Nobla (15.17 ± 1.22 individuals.m^−2^), a sand and rock substratum bay. The lowest values were in Moel y Don (0.87 ± 0.20 individuals.m^−2^), which had sand and rock substratum, and Moelfre Beach (4.07 ± 0.55 individuals.m^−2^), where the substratum was all rock. For *C. maenas*, the highest average abundance was reported at Moel y Don (0.70 ± 0.16 individuals.m^−2^, sand and rock) and Penmon Point (0.47 ± 0.14 individuals.m^−2^, stones and rock), and the lowest at Porth Trwyn (stones and rock), Porth-yr-Afon (stones and rock) and Cemlyn Bay (all rock). All had densities of 0.07 ± 0.05 individuals.m^−2^ (electronic supplementary material, table S2).

The average abundance of *A. equina* varied significantly among shores (Kruskal–Wallis, *H*_14_ = 55.35, *p* < 0.0001; electronic supplementary material, table S3*a*). Notably, Moel y Don was significantly different from all other shores (Wilcoxon rank sum, *p* < 0.05), and Porth Nobla was significantly different from all but two shores (Wilcoxon rank sum, *p* < 0.05). For *C. maenas*, average abundance also varied significantly among shores (Kruskal–Wallis*, H*_14_ = 55.35, *p* < 0.0001; electronic supplementary material, table S3*b*), and Moel y Don had the highest number of significant pairwise comparison differences (*n* = 10/14) to other shores (Wilcoxon rank sum, *p* < 0.05).

**Figure 2.**
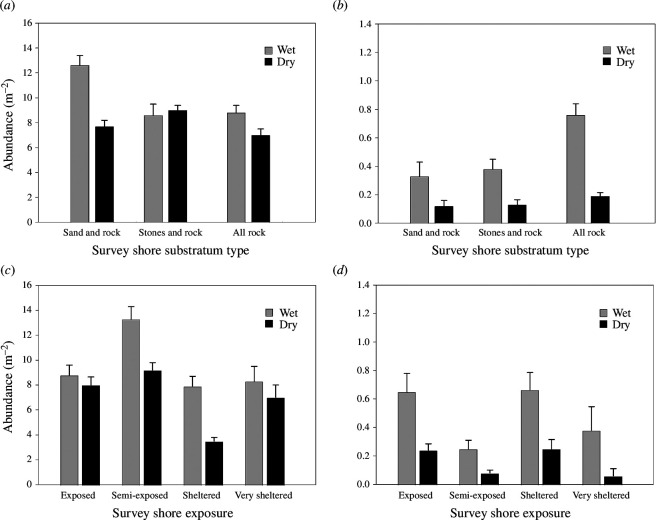
Average abundance (individuals.m^−2^ ± s.e.) of *Actinia equina* (*a*,*c*) and *C. maenas* (*b,d*) in relation to shores of different substratum type (sand and rock, stones and rock, all rock) and of varying exposures (following [24]). Wet and dry environments are also delimited (light grey bars and dark grey bars, respectively).

Average abundance of *A. equina* varied significantly among shore exposures (Kruskal–Wallis, *H*_3_ = 31.90, *p* < 0.0001; figure 2; electronic supplementary material, table S1), with all exposure comparisons showing significant differences in post hoc pairwise comparisons (Wilcoxon rank sum, *p* < 0.05), except between ‘semi-exposed’ and ‘very sheltered’ shores (Wilcoxon rank sum, *p* > 0.05). The average abundance of *C. maenas* also varied significantly among exposures (Kruskal–Wallis, *H*_3_ = 24.39, *p* < 0.0001; figure 2; electronic supplementary material, table S1), with post hoc testing identifying significant differences between ‘exposed’ and ‘semi-exposed’, and ‘semi-exposed’ and ‘sheltered’ shores (Wilcoxon rank sum, *p* < 0.05). In these comparisons, ‘exposed’ and ‘sheltered shores had higher predator abundances than ‘semi-exposed’ shores.

There was no difference in abundance of *A. equina* among substratum types (Kruskal–Wallis, *H*_2_ = 4.89, *p* = 0.09; figure 2; electronic supplementary material, table S1), but there was a difference for *C. maenas* (Kruskal–Wallis, *H*_2_ = 9.03, *p* = 0.01; figure 2; electronic supplementary material, table S1). Post hoc testing identified significant differences between ‘all rock’ and ‘sand and rock’, and ‘sand and rock’ and ‘stones and rock’ substratum types (Wilcoxon rank sum, *p* < 0.05). In both cases, ‘sand and rock’ abundance values were higher than for other substratum types.

Across all surveyed shores, *A. equina* average abundance varied significantly between wet and dry environments (Kruskal–Wallis, *H*_1_ = 38.59, *p* < 0.0001). *Actinia equina* was fairly evenly distributed between wet and dry sampled areas across the different survey shore substrata and exposures but was higher overall in wet environments on ‘sand and rock’ shores (12.28 ± 0.81 individuals.m^−2^) and ‘semi-exposed’ shores (12.99 ± 0.94 individuals.m^−2^) (figure 2). *C. maenas* abundance differed significantly between wet and dry environments across all survey shores (Kruskal–Wallis, *H*_1_ = 32.47, *p* < 0.0001). Abundance was greatest on ‘all rock’ shores (0.74 ± 0.10 individuals.m^−2^) and ‘exposed’ shores (0.63 ± 0.14 individuals.m^−2^) (figure 2). For both species, there was a statistically significant difference between wet and dry environments across all substratum types and exposures (Kruskal–Wallis, *p* < 0.05; electronic supplementary material, table S4), except for anemones on ‘very sheltered’ shores (electronic supplementary material, table S4).

**Figure 3.**
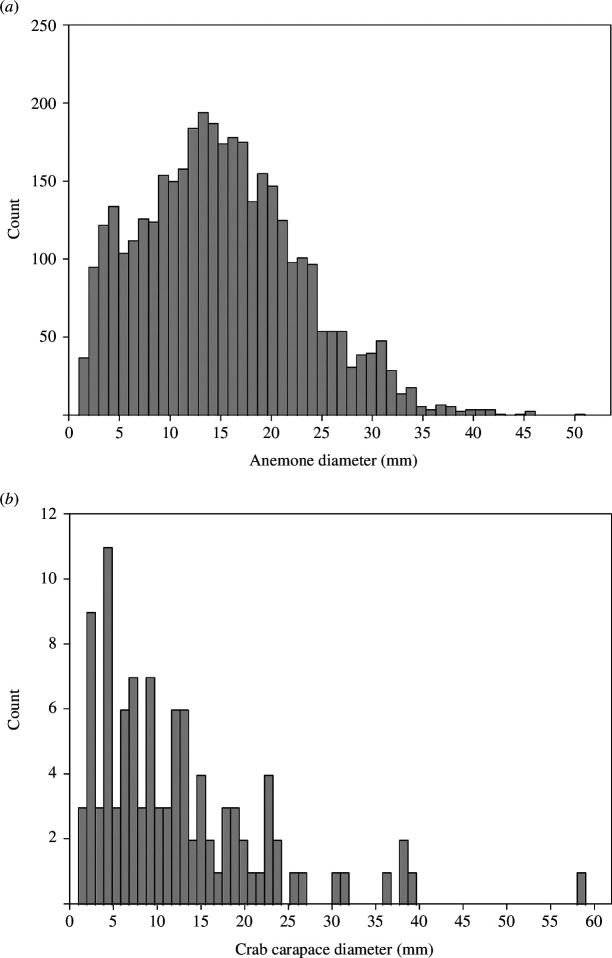
Overall size frequency distributions for populations around the Isle of Anglesey for (*a*) *Actinia equina*, where the total number of individuals measured was 14 772, and (*b*) *C. maenas*, where 416 crabs were measured.

Size frequency distributions for both species were right-skewed (figure 3), with the distribution for *C. maenas* being more strongly left skewed. Sizes ranged from 1 to 51 mm diameter (mean = 14.9 ± 1.0 mm) for *A. equina* and 1 to 59 mm (mean = 12.2 ± 0.9 mm) for *C. maenas*. Both distributions were dominated by small individuals, with small numbers of much larger specimens, as demonstrated by the median size for *C. maenas* at 10.0 mm, significantly less than the mean (one sample *t* = −2.4, *n* = 95, *p* < 0.018), whereas the median for *A. equina* (14.0 mm) was much closer to the mean and not significantly different from it.

For *A. equina,* individual, wet weight (g) and AFDM (g) were related to anemone diameter (mm), thus:

ln anemone wet weight = −5.30 + 2.01 ln anemone diameter (*F*_1,28_ = 98.34, *r* ^2^ = 0.78, *p* < 0.001),
ln anemone AFDM = −7.09 + 1.98 ln anemone diameter (*F*_1,28_ = 91.25, *r* ^2^ = 0.76, *p* < 0.001).

For *C. maenas* individuals, wet weight (g) and AFDM (g) were related to crab diameter (mm), thus:

ln crab wet weight = −4.47 + 1.98 ln crab diameter (*F*_1,28_ = 199.96, *r* ^2^ = 0.88, *p* < 0.001),
ln crab AFDM = −7.20 + 2.23 ln crab diameter (*F*_1,28_ = 223.54, *r* ^*2*^ = 0.89, *p* < 0.001).

From these relationships, the mean-sized *A. equina* in the beach surveys (12.2 mm) had a wet weight of 0.762 g (± 0.056 g) and an AFDM of 0.118 g (± 0.0009 g). The total anemone biomass across all shores (14 772 individuals) was 11 256 g (± 827 g) wet weight and 1743 g (± 13.3 g) AFDM. For *C. maenas*, the mean-sized individual (14.9 mm ± 1.0 mm) had a wet weight of 2.408 g (± 0.162 g) and an AFDM of 0.308 g (± 0.021 g). The total crab biomass across all shores (416 individuals) was 1002 g (± 67.6 g) wet weight and 128.1 g (± 8.74 g) AFDM.

### 3.2. Faecal egestion rates

**Figure 4.**
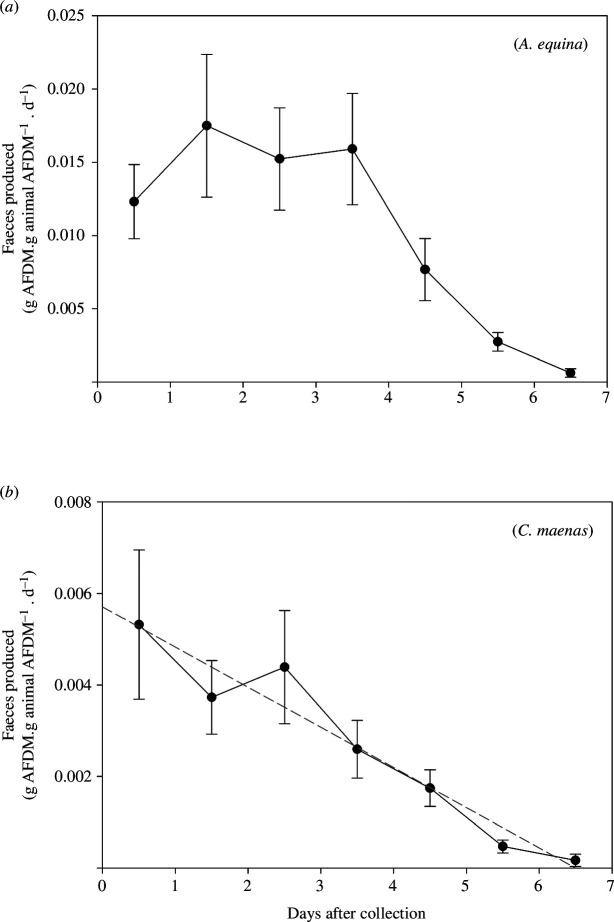
Faeces produced (g AFDM.g animal AFDM^−1^.d^−1^) with days after collection for (*a*), the anemone *A. equina* and (*b*), the crab *C. maenas*. Values shown are mean ± s.e. (*n* = 30). The two relationships are different with the anemone having similar production for 4 days followed by a decline and the crab showing a consistent decline in production from day 1. Because of this, only the relationship for *C. maenas* was regressed to produce a time-zero value (regression shown as grey dashed line). The regression was: crab faeces (g AFDM.g animal AFDM^−1^.d^−1^) = 0.00615 – 0.00088.day (*F*_1,208_ = 28.4, *r* ^2^ = 0.12, *p* < 0.001).

The pattern of FE in the days following collection differed in form between the two species (figure 4). In the anemone, *A. equina*, amounts of faeces produced remained at similar levels for 4 days before declining to levels close to zero by day 7. For the crab, *C. maenas*, there was a monotonic decrease in FE from day 1 until values were near zero, also on day 7. For both species, therefore, around one week is required to clear their digestive systems following feeding in natural conditions.

The aim of this research is to estimate the amounts of prey taken by populations of the two predators in the intertidal of the shores of the Isle of Anglesey. For this estimate, amounts of faeces produced at the time of collection are needed. To produce these estimates, different approaches are used for each species, because of the different forms of the relationship of faeces produced in the days following collection in the laboratory. For *A. equina*, the average of the first 4 days following collection is used because FE was similar across all 4 days (figure 4*a*). This produced a value for field FE of 0.0152 g AFDM.g animal AFDM^−1^.d^−1^ (± 0.0011). The day 1 value was lower than on days 2–4, and it is possible that there was an underestimate for the first 24 h because of either a small stress response on collection or faeces released early during collection. Average FE for days 2–4 was 0.0162 g AFDM.g animal AFDM^−1^.d^−1^ (± 0.0006), a value 6.6% higher. For *C. maenas,* because there was a monotonic decline in faecal production that started immediately after collection, a regression was used to calculate time-zero values (figure 4*b*). The time-zero value was 0.00571 g AFDM.g animal AFDM^−1^.d^−1^ (± 0.0004). FE on day 1 was 0.00532 (± 0.00163), a value 7.3% less.

### 3.3. Absorption efficiency, prey composition and prey consumed

AE was measured for three different prey types: the mussel, *M. edulis*, where excised soft tissues were offered to *C. maenas* and *A. equina*; the polychaete worm, *H. diversicolor*, offered as whole, or in portions of whole animal; and the shrimp, *C. crangon*, again offered whole or in portions of whole. All prey items were sourced from the same beaches as the predators under study. AEs differed between species with *C. maenas* producing AE values between 92.8% and 94% depending on prey species, whereas *A. equina* produced values between 40.5% and 95.8% (table 1). Overall means (means of means for different prey types) were 93.4% for *C. maenas* and 75.0% for *A. equina*, and these values were used to calculate prey consumed predation rates. This resulted in values of field food consumption (FE/AE) of 0.0216 g AFDM.g animal AFDM^−1^.d^−1^ for *A. equina* and 0.0061 g AFDM.g animal AFDM^−1^.d^−1^ for *C. maenas*.

**Table 1.** Absorption efficiencies for the crab *C. maenas* and the anemone *Actinia equina* fed three different prey types: soft tissues of the mussel *Mytilus edulis*; whole or portions of whole polychaete *Hediste diversicolor*; whole or portions of whole shrimp *Crangon crangon*.

predator species	prey type	mean AE (%)	s.e.	median AE (%)	max AE (%)	min AE (%)	*n*
*C. maenas*	*M. edulis* tissue	92.8	3.74	90.7	100	87.6	4
	*H. diversicolor*	93.4	1.43	94.5	95.5	89.2	4
	*Cr. crangon*	94.0	3.60	97.2	98.4	83.2	5
*A. equina*	*M. edulis* tissue	95.8	4.19	100	100	87.4	4
	*H. diversicolor*	40.5	17.3	36.4	72.3	12.7	4
	*Cr. crangon*	88.7	5.6	90.6	90.6	73.7	5

## 4. Discussion

The aim of this study was to estimate the amounts of prey taken by the dominant predators in the rocky intertidal of a typical temperate region, the Isle of Anglesey, north Wales, the anemone *A. equina* and the brachyuran crab *C. maneas*. For this, data were needed on the abundance and biomass of the populations of the two species combined with data on the amounts of faeces they produced, the AEs they exhibited when feeding on typical prey and the biomass characteristics of those prey.

### 4.1. Predator abundance and biomass

From detailed and extensive surveys of 15 beaches, there were 35 times as many anemones as crabs, but because the crabs were larger than the anemones, they were only 11.2 times greater in terms of wet (live) total weight of anemones across the shores of Anglesey and 13.6 times greater in AFDM. The difference in wet and AFDM comparisons reflects the loss of water on drying and from skeleton on ignition in the crab versus the loss of water on drying in the anemone.

The abundance of anemones on the shores surveyed ranged from 0–100 m^−2^ (mean = 8.21 ± 0.27, s.e.) is within the range of values reported for *A. equina* in the literature (electronic supplementary material, table S5), where values range from 0 to 345 m^−2^, with a mean of 45.8 m^−2^ (s.e. = 28.5) and a median of 3.9 m^−2^. The higher values have been reported from studies in the UK [31], but other UK studies have reported similar values to those here (e.g. [32]). Lower values have been reported from warmer parts of the species distribution, in the Mediterranean, where values are 1–6 m^−2^ [33,34], and in South Africa, where they are around 3 m^−2^ [35] The *A. equina* densities reported here are, therefore, representative of this species on temperate or cool temperate shores. Densities of other anemone species across the globe vary over a wider range, with means of 0.04–345 m^−2^ in the literature ([31,36]; electronic supplementary material, table S5). The sizes of anemones in our study were also similar to previous reports for this species in intertidal habitats in the UK [31,37]. The size range of anemones in this study was also within previously reported ranges for sites outside the UK (e.g. [34]; electronic supplementary material, table S5).

*C. maenas* abundance in this study ranged from 0 to 12 individuals m^−2^ (mean = 0.23 ± 0.02 s.e.). The overwhelming majority of published works on *C. maenas* distributions are based on individuals caught in traps (e.g. [38–40]). However, where data are available, density values range from 0.1 to 112 individuals m^−2^ (mean = 45.7, s.e. = 28.5; median = 3.8; *n* = 8), and two studies had mean values of less than 1, while five studies had values of less than 5 (electronic supplementary material, table S5). Thus, while the *C. maenas* densities reported in this study are low compared to others, they are within the range and not the lowest. *C. maenas* mean densities are higher than those reported for intertidal crab densities across the globe (mean = 35.2, s.e. = 15.4, median = 4.3). Excluding the *C. maenas* data, the mean density for other intertidal crabs is 25.6 (s.e. = 19.4) and the median is 2.6. This suggests that *C. maenas* occurs at similar but higher-than-average densities to intertidal crabs globally.

Large proportions of the *C. maenas* population on a shore migrate with the tide. The migrating proportion is predominantly mature individuals, whereas the static portion is mainly juveniles and smaller [40]. A study by Hunter & Naylor [41] collected between 0 and 8 *C. maenas* m^−2^ on a survey of shores in north Wales, similar to, but slightly lower than, this study. In traps, they caught between 5 and 38 crabs per 6 h of trap deployment, values similar to those found in trap-based surveys elsewhere. In their traps set at mid-tide level, over 50% of animals caught were less than 40 mm carapace width. The value was higher in higher shore locations and lower in lower shore locations. Aagard *et al*. [38] found that 33–45% of their catches in traps in Kerteminde fjord in Denmark were juveniles. This suggests our numbers probably account for around half the total crab population, but possibly the value could be as low as 25% or 30%. The populations of *A. equina* and *C. maenas* studied here were similar to those reported previously and were, therefore, representative of temperate populations in general. In terms of relative abundance of anemones and crabs, even allowing for a ×2 to ×4 underestimate of density owing to migrating crabs during tides, it is most likely that there were around 10 times as many anemones as crabs, as mean densities measured here indicated there were 35 times as many anemones as crabs on the beaches. Taking into account that juvenile crabs are smaller than adults, the biomass of *A. equina* was either similar to *C. maenas* biomass or up to twice the biomass.

### 4.2. Faecal egestion

The profiles of daily post-capture FE in this study differ substantially between *A. equina* and *C. maenas*. In the anemone, production of faeces remained constant for 4 days; in the crab, amounts produced declined monotonically from day 1; and in both species, faecal production was close to zero after 7 days. This difference led to the use of a different method to calculate field faecal production rates. The differences in profiles probably reflect the feeding mechanisms of the two species. From the profiles, we can infer that it takes the anemones between 4 and 7 days to complete digestion of a meal and that the crab has a continuous throughput of food to faeces that declines gradually when no more food is available and requires around 7 days for the gut to clear completely.

The anemone *A. equina* is a batch feeder, consuming prey whole, digesting materials from around skeletal components and then ejecting undigested soft tissue elements alongside skeletal material. It has a blind-ended digestive system, the gastrovascular cavity that performs the functions of both a mouth and anus and faecal material is ejected through it. Once full, no more food can be added until the previous meal is digested and remains have been ejected. The crab, *C. maenas,* on the other hand, has a double-ended digestive system with separate mouth and anus. It feeds continuously by picking soft tissue from skeletons, and it does not ingest skeletal material. Because of this, AFDM was used to compare the amounts of faeces produced, because this measures only organic, non-skeletal production.

The rate of production of faeces per gram of animal organic content in *A. equina* (0.0152 g AFDM.g animal AFDM^−1^.d^−1^, s.e. = 0.0011) was 2.66 times higher than the value for *C. maenas*. If the comparisons are done on a dry weight basis, then the anemone values were 2.17 times higher than the crab. FE has been used in the past to investigate the seasonality of feeding and to assess if feeding ceases in winter periods in a range of marine species including predators and scavengers (e.g. [13,27–29]).

### 4.3. Absorption efficiency

AE was measured for each predator species using different prey types. When both species were fed just the soft tissues of *M. edulis*, absorption levels were very high (93% and 96%; table 1), as would be expected from predators consuming purely organic tissue [42–44], or when absorption is measured on an organic or energy basis (e.g. [45]), but where predators consume prey with substantial amounts of skeleton, AE values are much lower (e.g. [46]). The average values here for *A. equina* are also similar to those reported for the anemone *Anthopleura elegantissima* when fed live *Artemia nauplii* [47].

The relatively large differences in AE between the two species studied here when fed the polychaete *H. diversicolor* or the shrimp *Cr. crangon* can be explained by the different feeding mechanisms used by *C. maenas* and *A. equina. C. maenas* uses its chelae to pick soft tissue from prey items it catches. It thus does not consume indigestible material such as skeletons. *Actinia equina*, on the other hand consumes prey whole and digests soft tissues in its gastrovascular cavity with the undigested skeletal material being ejected as part of the faeces. Crustacean skeletons account for around 25% to over 50% of the dry weight of the animals, with shrimp species having lower proportions than portunid crabs and brachyuran crabs having the highest values [48]. Faecal mass estimates need, therefore, to be adjusted for the apportionment of skeletal material before calculating the amounts of prey consumed.

### 4.4. Estimating predation rates

The density data presented here show that there were up to 10 times as many anemones in the intertidal as crabs and that biomass was between similar and two times more in anemones than crabs. Faecal production rates were estimated at 2.66 times higher per unit biomass in anemones than in crabs using AFDM as the metric. Including AE values raised that to 3.54 times higher. The difference between just using FE and incorporating AE accounts for differences in the consumption of skeletal material. In the feeding trial, *C. maenas* picked soft tissue from skeletons and did not ingest skeletons, whereas *A. equina* did consume skeletal material. Thus, our data suggest that on the shores of the Isle of Anglesey, around 3.54–7.08 times as much biomass is consumed per day by anemones as by crabs, depending on the density values used, and that the common soft-bodied predator, *A. equina*, has a substantially larger predation impact than the durophagous *C. maenas*. These estimates are perforce rough, but they do highlight the often ignored, or at least undervalued, impact of soft-bodied predators. Predation pattern hypotheses have, in the past, been predominantly erected around observations of durophagous predators and grazing mobile fishes, and these have supported the hypothesis that predation levels are high in low latitudes and low at high latitudes (e.g. [10,11,49]).

Densities of anemones in Antarctic sediments have been reported to be as high as 1600 m^−2^ at Rothera Point [50] and 2300 m^−2^ at McMurdo Sound [51], and while the very large species inhabiting hard substrata are much less dense, they do occur in densities in excess of 12 m^−2^ and biomasses over 1 kg live weight m^−2^ ([52] and L. S. Peck, personal observation, 1997, 1998, 2002, 2005, 2006, 2008, 2010, 2014, 2016, 2019). They, therefore, very likely exert a large local predation pressure and suggest that predation in areas without reptant decapods might not be as low as previously thought.

We are not aware of FE and AEs being used in the past to estimate food consumption and predation rates in a marine ecological setting.

### 5. Conclusions

In this analysis of two of the most common predators on temperate intertidal shores of the Isle of Anglesey, UK, levels of predation were up to seven times higher from the anemone *A. equina* compared to the reptant decapod *C. maenas*. The main factors underlying this outcome were the higher density of anemones on the shores and the higher feeding rates of anemones per unit biomass. Even taking into account that *C. maenas* densities elsewhere are often higher, the suggestion is that anemone predation is as high as, if not higher than, crab predation on temperate intertidal shores. The implications of this work are that estimates of predation in marine benthic food webs and perceived trends in predation pressure need to be re-assessed, incorporating the impacts of soft-bodied predators.

## Data Availability

The data have been deposited with Dryad [53]. Supplementary material is available online [54].
